# Supplementary material for: Policy shaping based on the learned preferences of others accounts for risky decision-making under social observation
Source: eLife. 2025 Sep 12;13:RP102228. doi: 10.7554/eLife.102228 (PMC12431773; doi:10.7554/eLife.102228)
Supplement: Supplementary file 1. [file elife-102228-supp1.pdf]

Supplementary File 1

Title:
Policy shaping based on the learned preferences of others accounts for risky decision-making under social observation

Authors:
HeeYoung Seon[1], Dongil Chung[*1]

Affiliations:
[1]Department of Biomedical Engineering, Ulsan National Institute of Science and Technology, Ulsan, South Korea

*Correspondence should be made to
DC (dchung@unist.ac.kr; +82-52-217-2744; 50 UNIST-gil, Ulsan 44919, South Korea)

Table A. Demographic data

	n = 43
Male/Female (% female)	25/18 (41.86%)
Age	21.28 ± 2.37
Education (%)	
High school graduated	1 (2.33%)
Enrolling University	41 (95.35%)
Advanced degree (Enrolling or graduated)	1 (2.33%)
Monthly income	
< 1,000	4 (9.30%)
1,000 - 2,000	1 (2.33%)
2,000 - 3,000	2 (4.65%)
3,000 - 4,000	8 (18.60%)
4,000 - 5,000	6 (13.95%)
5,000 - 6,000	5 (11.63%)
6,000 - 7,000	6 (13.95%)
7,000 - 8,000	5 (11.63%)
8,000 - 9,000	4 (9.30%)
9,000 - 10,000	0 (0%)
10,000<	2 (4.65%)

Values are expressed as means ± SD unless noted otherwise. A monthly income was converted from Korean Won to U.S Dollars (1,000 Korean Won is approximately equivalent to 1 U.S Dollar).

Table B. Regions showing significant associations with final decision probabilities P(chosen) during the Solo phase

Positive responses

		MNI coordinates					
Region	Laterality	x	y	z	Cluster size k_E	t	Z
Ventromedial prefrontal cortex **	L	-3	62	-13	99	5	4.21
Ventral striatum *	R	3	14	-10	9	3.97	3.52
Hippocampus	L	-27	-13	-16	24	4.83	4.11
Posterior Cingulate gyrus	R	12	-58	23	13	4.2	3.68
	L	-9	-58	20	46	4.52	3.9
Middle Temporal Gyrus	L	-51	-76	20	8	4.08	3.6
Fusiform gyrus	R	45	-22	-19	5	4.02	3.55
Insula	R	48	-4	8	6	3.82	3.41

Height threshold: $t = 3.25$; extent threshold: $k_E = 5$ voxels; L: left; R: right; clusters are thresholded at $P < 0.001$; *cluster-level $P_{FWE, SVC} < 0.05$; **cluster-level $P_{FWE, SVC} < 0.01$.

Negative responses

		MNI coordinates					
Region	Laterality	x	y	z	Cluster size k_E	t	Z
Dorsal anterior cingulate cortex **	R	12	32	29	118	5.22	4.35
Superior Frontal Gyrus	R	18	23	50	17	4.1	3.61
Insula	R	30	23	2	11	3.81	3.41

Height threshold: $t = 3.25$; extent threshold: $k_E = 5$ voxels; L: left; R: right; clusters are thresholded at $P < 0.001$; **cluster-level $P_{FWE, SVC} < 0.01$.

Table C. Regions showing significant associations with subjective utility differences between the chosen and unchosen options during the Solo phase

Positive response

		MNI coordinates					
Region	Laterality	x	y	z	Cluster size k_E	t	Z
Ventromedial prefrontal cortex		0	65	-7	27	4.55	3.92
Inferior frontal gyrus	L	-48	-1	8	8	4.54	3.91
Inferior parietal lobule	L	-42	-82	26	15	4.44	3.84
Precuneus	R	6	-49	35	10	3.76	3.37
	L	-15	-61	23	31	4.14	3.64
Hippocampus	L	-30	-10	-16	11	4.05	3.58

Height threshold: t = 3.25; extent threshold: k_E = 5 voxels; L: left; R: right; clusters are thresholded at $P < 0.001$.

Negative response

		MNI coordinates					
Region	Laterality	x	y	z	Cluster size k_E	t	Z
Dorsal anterior cingulate cortex ***	L	-6	26	32	301	7.71	5.64
Occipital Lobe	L	-24	-79	-1	64	5.75	4.66
	R	18	-94	-4	29	4.12	3.62
Postcentral gyrus	R	57	-28	53	58	5.43	4.47
Dorsolateral prefrontal cortex	L	-39	47	11	60	4.88	4.14
Inferior frontal gyrus	L	-48	2	23	7	4.12	3.63
	L	-33	-55	47	8	3.67	3.3
Cerebellum	R	42	-61	-37	32	4.6	3.96
Inferior parietal lobule	L	-42	-52	-40	45	4.54	3.91

Height threshold: t = 3.25; extent threshold: k_E = 5 voxels; L: left; R: right; clusters are thresholded at $P < 0.001$; ***cluster-level $P_{FWE, SVC} < 0.001$.

Table D. Regions showing significant associations with final decision probabilities P(chosen) during the Observed phase

Positive responses

		MNI coordinates					
Region	Laterality	x	y	z	Cluster size k_E	t	Z
Temporoparietal junction	R *	63	-40	17	148	4.85	4.11
	L	-54	-37	14	64	4.49	3.88
Striatum *	R	6	14	-7	18	4.51	3.9
Amygdala *	L	-21	-13	-19	13	4.74	4.05
Posterior Cingulate gyrus	L	-15	-34	41	12	4.35	3.79
Precentral Gyrus	R	54	-1	8	9	4.26	3.72
Superior temporal gyrus	R	51	-19	-7	8	3.68	3.3
Insula	R	42	-7	14	5	3.65	3.28

Height threshold: t = 3.25; extent threshold: k_E = 5 voxels; L: left; R: right; clusters are thresholded at $P < 0.001$; *cluster-level $P_{FWE, SVC} < 0.05$.

Negative responses

		MNI coordinates					
Region	Laterality	x	y	z	Cluster size k_E	t	Z
Dorsal anterior cingulate cortex ***	L	-6	14	53	275	5.33	4.42
Inferior frontal gyrus	R	48	11	26	84	5.53	4.53
	L	-48	5	32	99	4.72	4.03
Insula	R	30	26	-4	60	4.66	3.99
	L	-27	26	-10	18	5.34	4.42
	L	-33	14	-4	5	3.68	3.3
ventrolateral prefrontal cortex	R	42	50	8	81	4.32	3.76
	L	-45	53	-1	225	5.29	4.39
Inferior parietal lobule	R	48	-43	59	376	5.11	4.28
	L	-36	-52	47	290	4.82	4.1
Middle Occipital Gyrus	R	39	-91	2	182	4.94	4.17
Orbitofrontal cortex	R	21	35	-25	36	4.86	4.12
	L	-15	41	-16	13	4.49	3.88
	L	-18	65	-4	5	4.02	3.55
Superior parietal gyrus	R	33	-82	38	23	4.18	3.67
	R	33	-70	29	7	3.6	3.25
Superior occipital gyrus	L	-6	-103	-7	52	4.05	3.57
Cingulate gyrus	L	-3	-16	29	16	3.96	3.51
Fusiform Gyrus *	R	60	-46	-22	21	3.96	3.51
Middle frontal gyrus	L	-36	11	62	6	3.55	3.21
Cerebellum	L	-39	-67	-16	71	4.23	3.71
	L	-51	-70	-31	8	3.87	3.45
	R	3	-52	-34	6	3.84	3.42

Height threshold: t = 3.25; extent threshold: k_E = 5 voxels; L: left; R: right; clusters are thresholded at $P < 0.001$; *cluster-level $P_{FWE, SVC} < 0.05$, ***cluster-level $P_{FWE, SVC} < 0.001$.

Table E. Regions showing significantly higher responses during Observer trials compared to No observer trials at the time which the identity of the observer was revealed

Positive responses

		MNI coordinates					
Region	Laterality	x	y	z	Cluster size k_E	t	Z
Medial prefrontal cortex	L	-3	50	14	22	3.18	2.92
Occipital lobe	L	-21	-100	-1	3194	11.55	7.02
Insula	R	27	14	11	56	4.41	3.82
Superior frontal gyrus	L	-9	59	26	85	3.93	3.49
Hippocampus	L	-21	-34	-4	50	3.86	3.44
Cingulate gyrus	R	9	-7	32	35	3.63	3.27
	L	-6	8	50	11	3.03	2.8
Inferior frontal gyrus	L	-39	35	-10	112	3.63	3.27
Putamen	L	-30	-1	8	122	3.56	3.21
Temporal pole	R	30	20	-40	42	3.5	3.17
Postcentral gyrus	L	-36	-28	41	19	3.36	3.06
	L	-39	-43	65	20	3.28	3
Supra marginal gyrus	R	63	-22	32	44	3.34	3.04
Ventrolateral prefrontal cortex	L	-42	50	2	27	3.32	3.03
Orbitofrontal cortex	L	-18	35	-28	19	3.26	2.98
Amygdala	R	**24**	**-4**	**-13**	19	3.1	2.86
Medial frontal gyrus	L	-39	-4	56	12	3.09	2.85
Precentral gyrus	R	60	5	38	15	3.05	2.82
Middle temporal gyrus	L	-57	-7	-19	15	3.05	2.82
Cerebellum	R	0	-55	-28	11	3.01	2.78
Midbrain	L	-12	-16	-13	16	3.51	3.18

Height threshold: $t = 2.76$; extent threshold: $k_E = 10$ voxels; L: left; R: right; clusters are thresholded at $P < 0.005$.

Table F. Regions showing significant associations between $dmPFC_{contrast}$-$dmPFC_{PPI}$ connectivity and individuals' log-transformed Social reliance

Positive response

		MNI coordinates					
Region	Laterality	x	y	z	Cluster size k_E	t	Z
Medial prefrontal Cortex *	R	3	50	5	17	3.92	3.47
Temporoparietal junction *	L	-66	-49	8	7	3.93	3.48
Inferior frontal gyrus	R	54	23	-1	47	4.82	4.08
	L	-51	32	5	30	4.27	3.72
Middle frontal gyrus	R	30	59	2	48	4.76	4.04
	R	36	32	29	51	4.55	3.9
Middle Temporal Gyrus	L	-60	-46	-16	59	4.59	3.93
Putamen	R	24	-4	5	22	4.41	3.81
Postcentral gyrus	R	42	-10	41	23	4.22	3.68
Superior temporal sulcus	R	66	-16	-7	15	4.17	3.65
Inferior parietal lobule	R	39	-76	38	13	4.14	3.63
	R	39	-55	35	11	3.88	3.44
	L	-33	-70	41	6	3.6	3.23
Superior frontal gyrus	R	27	26	53	18	4	3.53
Dorsal anterior cingulate cortex	R	6	32	32	27	3.95	3.49
Superior parietal gyrus	L	-33	-55	56	9	3.93	3.47
Inferior fontal gyrus	R	54	5	23	13	3.74	3.34
Precuneus	L	-15	-73	41	6	3.72	3.32
Amygdala *	L	-27	2	-4	11	3.68	3.3
Hippocampus	L	-33	-25	-4	6	3.57	3.21
Supplementary motor area		0	20	65	8	3.53	3.18
Cerebellum	L	-30	-76	-37	15	3.81	3.39

Height threshold: t = 3.25; extent threshold: k_E = 5 voxels; L: left; R: right; clusters are thresholded at $P < 0.001$; *cluster-level $P_{FWE,\ SVC} < 0.05$.

Table G. First 30 sets of gambles used for the first phase ('Solo') and the third phase ('Observed') of the task

	Safe option	Risky option		
	Payoff	High payoff	Probability	ΔEV (Risky – Safe)
1	25	15	0.5	-17.5
2	70	20	0.75	-55
3	10	25	0.25	-3.75
4	5	25	0.25	1.25
5	3	25	0.25	3.25
6	14	25	0.5	-1.5
7	11	25	0.5	1.5
8	9	25	0.5	3.5
9	23	25	0.75	-4.25
10	15	25	0.75	3.75
11	5	25	0.75	13.75
12	10	48	0.25	2
13	6	48	0.25	6
14	30	48	0.5	-6
15	23	48	0.5	1
16	18	48	0.5	6
17	42	48	0.75	-6
18	31	48	0.75	5
19	25	48	0.75	11
20	14	48	0.75	22
21	12	76	0.5	26
22	20	78	0.75	38.5
23	47	90	0.25	-24.5
24	31	90	0.25	-8.5
25	20	90	0.25	2.5
26	53	90	0.5	-8
27	43	90	0.5	2
28	37	90	0.5	8
29	76	90	0.75	-8.5
30	59	90	0.75	8.5

Table H. Second 30 sets of gambles used for the second phase ('Learning') of the task

	Safe option	Risky option		
	Payoff	High payoff	Probability	ΔEV (Risky – Safe)
1	11	14	0.5	-4
2	3	15	0.25	0.75
3	6	15	0.5	1.5
4	25	18	0.75	-11.5
5	14	20	0.75	1
6	20	23	0.5	-8.5
7	13	23	0.5	-1.5
8	3	23	0.5	8.5
9	12	23	0.75	5.25
10	15	34	0.25	-6.5
11	6	34	0.25	2.5
12	23	34	0.5	-6
13	32	34	0.75	-6.5
14	17	34	0.75	8.5
15	20	48	0.5	4
16	29	53	0.5	-2.5
17	24	53	0.5	2.5
18	38	53	0.75	1.75
19	20	53	0.75	19.75
20	15	58	0.25	-0.5
21	10	58	0.25	4.5
22	48	58	0.75	-4.5
23	90	78	0.5	-51
24	60	78	0.75	-1.5
25	25	90	0.25	-2.5
26	21	90	0.25	1.5
27	15	90	0.25	7.5
28	38	90	0.5	7
29	64	90	0.75	3.5
30	55	90	0.75	12.5

Table I. Questions about individuals' impressions of each predicted partner

Questions	Mean ± SD		
	Risk-seeking	Risk-averse	Difference (Seeking - Averse)
Q1. I like the way this partner chooses.	1.63±5.82	2.93±5.51	-1.30±8.73
Q2. I trust this partner.	-0.21±4.41	5.02±3.65	-5.23±5.58
Q3. This partner prefers risk.	6.86±2.07	-8.33±1.52	15.19±2.78
Q4. This partner is attractive.	5.07±2.83	0.86±4.33	4.21±5.29
Q5. This partner is well aware of his or her preferences.	5.42±3.70	5.67±3.77	-0.26±4.52
Q6. This partner has good academic grades.	2.21±3.95	4.49±3.07	-2.28±4.92
Q7. This partner made consistent choices.	4.98±3.62	5.12±4.06	-0.14±4.92
Q8. This partner has a similar preference to mine.	-0.26±5.83	2.77±5.88	-3.02±10.36
Q9. Answer "5".	5.00±0	5.05±0.49	-0.05±0.49

Supplementary References

Dohmen, T., Falk, A., Huffman, D., Sunde, U., Schupp, J., & Wagner, G. G. (2011). Individual risk attitudes: Measurement, determinants, and behavioral consequences. *Journal of the european economic association*, *9*(3), 522-550.

Kang, J. (2013). A study on the individual differences in empathy: Using emotional priming. *Unpublished master's dissertation). Korea University, Seoul, South Korea*.

Kim, C.-G., Kim, J. S., Jung, J.-G., Kim, S.-S., Yoon, S.-J., & Suh, H.-S. (2014). Reliability and validity of alcohol use disorder dentification test-Korean revised version for screening at-risk drinking and alcohol use disorders. *Korean journal of family medicine*, *35*(1), 2.

Lee, E.-H., Lee, S.-J., Hwang, S.-T., Hong, S.-H., & Kim, J.-H. (2017). Reliability and validity of the Beck Depression Inventory-II among Korean adolescents. *Psychiatry investigation*, *14*(1), 30.

Mehrabian, A., & Stefl, C. A. (1995). Basic temperament components of loneliness, shyness, and conformity. *Social Behavior and Personality: an international journal*, *23*(3), 253-263.

Rotter, J. B. (1967). A new scale for the measurement of interpersonal trust. *Journal of personality*.

Steinberg, L., & Monahan, K. C. (2007). Age differences in resistance to peer influence. *Developmental psychology*, *43*(6), 1531.
